# Hot-Band-Absorption-Induced Anti-Stokes Fluorescence of Aggregation-Induced Emission Dots and the Influence on the Nonlinear Optical Effect

**DOI:** 10.3390/bios11110468

**Published:** 2021-11-22

**Authors:** Yuhuang Zhang, Jing Zhou, Shiyi Peng, Wenbin Yu, Xiaoxiao Fan, Wen Liu, Zikang Ye, Ji Qi, Zhe Feng, Jun Qian

**Affiliations:** 1 State Key Laboratory of Modern Optical Instrumentations, Centre for Optical and Electromagnetic Research, College of Optical Science and Engineering, International Research Center for Advanced Photonics, Zhejiang University, Hangzhou 310058, China; yuhuangzhang@zju.edu.cn (Y.Z.); zhoujing_bmo@zju.edu.cn (J.Z.); 22030026@zju.edu.cn (S.P.); 21630074@zju.edu.cn (W.Y.); fanxx_gs@zju.edu.cn (X.F.); zhefeng@zju.edu.cn (Z.F.); 2 Key Laboratory of Optical Information Detecting and Display Technology, Zhejiang Normal University, Jinhua 321004, China; 3 Department of Chemistry, Zhejiang University, Hangzhou 310027, China; zikangye@zju.edu.cn; 4 State Key Laboratory of Medicinal Chemical Biology, Key Laboratory of Bioactive Materials, Ministry of Education, College of Life Sciences, Nankai University, Tianjin 300071, China; qiji@nankai.edu.cn

**Keywords:** anti-Stokes fluorescence, aggregation-induced emission, hot-band absorption, in vivo confocal imaging, multi-photon microscopy

## Abstract

Hot-band absorption (HBA)-induced anti-Stokes fluorescence (ASF) with longer-wavelength excitation is one effective pathway to deep penetration and low autofluorescence in intravital fluorescence imaging, raising demands for fluorophores with broad spectra, high absorption, and strong emission. However, typical fluorescent dyes display some emission quenching when their concentration is increased in order to obtain brighter fluorescence. In this work, the HBA-induced ASF of aggregation-induced emission (AIE) dots is reported. BPN-BBTD dots were synthesized and confirmed with a fluorescence enhancement and a considerable ASF intensity. In addition, the mechanism of ASF and the HBA process of BPN-BBTD dots were carefully validated and discussed. To obtain the full advantages of the long-wavelength excitation and the short fluorescence lifetime in deep-tissue bioimaging, a large-depth ASF confocal microscopic imaging of in vivo cerebral vasculature was conducted under the excitation of a 980 nm continuous wave laser after intravenous injection of BPN-BBTD dots. Meanwhile, the 3D structure of the cerebrovascular network was successfully reconstructed.

## 1. Introduction

Fluorescence imaging is a powerful tool for deep-penetration bioimaging due to its excellent resolution [1,2]. In contrast to the typical Stokes luminescence, anti-Stokes luminescence utilizes long-wavelength excitation light to emit short-wavelength photons which need additional energy. Long excitation wavelengths, such as in the second near-infrared window (NIR-II, 900–1880 nm), are well known for their large penetration depth, low photo-damage, and low autofluorescence in biological tissues compared to visible excitation [3,4,5,6,7,8,9,10]. In addition, near-infrared excitation has lower scattering, resulting in finer focal spots and higher-quality images in deep-tissue confocal imaging [3,11,12]. Thus, these unique optical properties endow anti-Stokes luminescence with significant advantages in large-depth confocal imaging.

The phenomena of anti-Stokes luminescence can be divided into two broad categories according to different sources of additional energy [13,14]. One source of additional energy is another excitation photon, as in the multi-photon absorption process and upconversion processes based on lanthanide or triplet–triplet annihilation [5,9,15,16,17]. Thermal photons are another source, as in the hot-band absorption (HBA) process and the thermally activated delayed fluorescence (TADF) process [14,18,19]. All these processes have been successfully utilized in in vivo bioimaging, while they all have certain different limitations. The multi-photon absorption process has been widely utilized in two/three-photon microscopy. However, it commonly requires femtosecond (fs) lasers to provide high-peak-intensity excitation which is costly [20]. The bio-applications of the upconversion process are restricted by the small absorption cross-section and heavy metal ions for lanthanide-based upconversion, and the low photostability for triplet–triplet-annihilation-based upconversion [13,20,21,22]. The processes of thermally activated delayed fluorescence and HBA have drawn interest in recent years because they can be observed in organic dyes with high absorption [6,14,23]. In general, the process of anti-Stokes thermally activated delayed fluorescence is similar to the process of HBA-induced anti-Stokes fluorescence. However, the former has a long lifetime on the order of milliseconds to microseconds due to the involvement of the triplet states, in comparison with the short lifetime of the latter [14,24,25,26,27,28]. Because of its short fluorescence lifetime, the HBA process is suitable for fast-scanning imaging such as fluorescence confocal microscopy. Fluorescence confocal microscopy achieves high spatial resolution by placing pinholes or collimators to restrict the luminescence and detection region [29]. If fast scanning is performed, the integration time of a single pixel could be as low as 10 μs, which is not suitable for a long-lifetime luminescence dye [3]. However, the current organic HBA-induced anti-Stokes fluorescence dyes in use, such as indocyanine green (ICG), suffer from aggregation-induced quenching which hinders further improvement of brightness [14]. Fortunately, aggregation-induced emission (AIE) dyes were first proposed in 2001, which avoided the aggregation-caused quenching effect and emitted bright fluorescence in the aggregate or solid state [30]. When the concentration increase, fluorescence is always enhanced [31]. Thus, it could be an excellent strategy to combine AIE dyes with the HBA process in bioimaging.

In this work, the anti-Stokes fluorescence based on the HBA process is reported in AIE dyes. We prove that BPN-BBTD dyes have typical AIE characteristics, considerable anti-Stokes fluorescence intensity, and excellent photostability. By measuring the power dependence, lifetime, and temperature dependence of anti-Stokes fluorescence, the anti-Stokes fluorescence process was carefully determined to be the HBA process. Furthermore, considering the two absorption peaks at 363 nm and 706 nm of BPN-BBTD dyes, BPN-BBTD may have both single-photon absorption and two-photon absorption with femtosecond laser excitation. To explore the competitive relationship between HBA and multi-photon absorption, power dependence measurements of BPN-BBTD dots were performed and we found that the nonlinear effect might be weakened due to the increased HBA. In addition, to exert the long-wavelength excitation and short fluorescence lifetime in deep-tissue bioimaging, anti-Stokes fluorescence confocal microscopy imaging of the cerebral vasculature in a mouse model was conducted with the largest depth of ~450 μm below the skull under 980 nm excitation. The 3D structure of the cerebrovascular network was successfully reconstructed. We believe that our work can provide a reference for future research about HBA-induced anti-Stokes fluorescence confocal microscopy and nonlinear microscopy in deep tissue.

## 2. Materials and Methods

### 2.1. Chemicals and Materials

Chloroform, Pluronic F127, and tetrahydrofuran (THF) were purchased from Sigma-Aldrich. Deionized water was used in all experiments. BPN-BBTD was synthesized in our laboratory as per the procedure in our previous publication [32].

### 2.2. Fabrication of BPN-BBTD Dots

Briefly, 3 mg BPN-BBTD in 3 mL chloroform was blended with 36 mg Pluronic F-127 in 3 mL chloroform and sonicated for 5 min to get a uniform solution. Then, the mixture was evaporated in a rotating round-bottom flask until dry under vacuum at room temperature. Next, 1.5 mL deionized water was added to the residue and sonicated for 5 min until the residue was dissolved completely and formed a clear aqueous dispersion. Finally, the aqueous dispersion of BPN-BBTD dots was purified with a 0.45 μm syringe filter.

### 2.3. Absorption and Fluorescence Spectra Measurement

The absorption and fluorescence spectra of BPN-BBTD dots were measured respectively by a UV-Visible spectrophotometer (measurement wavelength range 190–900 nm, UV-2550, Shimadzu, Japan) and a lab-built system for the measurement of fluorescence spectra based on a spectrometer (PG2000, Ideaoptics, Shanghai, China). To measure the Stokes fluorescence spectra, a 665 nm continuous-wave (CW) semiconductor laser was used as the excitation light and a 700 nm short-pass filter (FESH0700, Thorlabs, Newton, NJ, USA) was placed in the path of the excitation light to filter out the long-wavelength wing. The excitation beam was focused on the aqueous dispersion of BPN-BBTD dots in a quartz cell through a lens. Of note, to reduce the self-absorption of the Stokes fluorescence, the excitation light was focused near the border of the quartz cell. The Stokes fluorescence was collected by an objective (25×/1.05, Olympus, Japan) and a 700 nm long-pass filter (FELH0700, Thorlabs, Newton, NJ, USA), and finally detected by the spectrometer. To measure the anti-Stokes fluorescence spectra, we replaced the 665 nm laser, the 700 nm short-pass filter, and the 700 nm long-pass filter with a 980 nm laser, a 900 nm long-pass filter (FELH0900, Thorlabs, Newton, NJ, USA), and a 900 nm short-pass filter (FESH0900, Thorlabs, Newton, NJ, USA), respectively. In addition, the quartz cell was placed on a thermostatic table to evaluate fluorescence spectra at different temperatures.

### 2.4. Animal Preparation for Cerebrovascular Microscopic Imaging

Institute of Cancer Research (ICR) mice (female, 6 weeks old) were used for in vivo experiments. They were provided by the Zhejiang Academy of Medical Sciences and raised at the Experimental Animal Center of Zhejiang University. The room temperature of the rearing environment was maintained at 24 °C with a 12 h light/dark cycle. Mice were continuously supplied with water and standard laboratory chows. All the animal procedures were conducted in accordance with “The National Regulation of China for Care and Use of Laboratory Animals” and supported by the Institutional Ethical Committee of Animal Experimentation of Zhejiang University. After anesthesia, the skull of the mouse was partly removed via microsurgery. Next, the hole in the skull was covered with a round thin glass to protect the brain, and a small metal ring was attached to the skull with dental cement. The mouse was fixed on a mouse rack to stabilize its head. Then, the aqueous solution of BPN-BBTD dots (2 mg/mL, 200 μL) was intravenously injected.

### 2.5. Optical Setup of the First Near-Infrared (NIR-I, 760–900 nm) Anti-Stokes Fluorescence Confocal Microscopy

Briefly, a 980 nm continuous-wave semiconductor laser was collimated and reflected by a 900 nm short-pass dichroic mirror (DMSP900R, Thorlabs, Newton, NJ, USA), and introduced into a 2-axis (X and Y) scanning galvanometer system, which deflected the beam to scan. Then, the excitation light passed through the scan lens, tube lens, and objective lens (25×/1.05, Olympus, Japan), and was finally focused on the sample. The anti-Stokes fluorescence emitted by the sample was collected by the same objective and passed back through the same optical path. The anti-Stokes fluorescence then passed through the 900 nm short-pass dichroic mirror, a 700 nm long-pass filter, and a 900 nm short-pass filter to remove the excitation light. A collimator was set to couple the anti-Stokes fluorescence into a fiber as a pinhole (core diameter = 1 μm). The optical signal propagated along the fiber to the detection plane of a PMT (H7422-50, Hamamatsu, Japan). Current signals were converted into digital signals to reconstruct images on the computer.

### 2.6. Power Dependence Measurement at Different Temperatures

In this measurement, we used a lab-built measuring system to explore the relationship between the anti-Stokes fluorescence intensity, excitation power, and temperature. Samples in the quartz cell were heated and kept at different temperatures with a thermostat. A thermal imager (TiS20, Fluke, Everett, WA, USA) was applied to accurately monitor the temperature of samples. A tunable femtosecond laser (80 MHz, Coherent Chameleon Ti: Sapphire, USA) and a tunable continuous-wavelength laser (Matisse, Spectra-Physics, Milpitas, CA, USA) were utilized to provide excitation wavelengths at 980 nm. The collimated excitation beam was reflected by a 900 nm short-pass dichroic mirror and focused via a high-numerical-aperture objective (25×/1.05, Olympus, Japan) on the samples. The anti-Stokes fluorescence signals were collected by the same objective and passed through the same dichroic mirror to remove excitation photons. After passing through a 700 nm long-pass filter and a 900 nm short-pass filter, the anti-Stokes fluorescence was focused by a lens and detected by the PMT in sequence. An amplifier (C12419, Hamamatsu, Japan) was used to convert current signals generated by the PMT to voltage signals. Finally, a NI data acquisition card (USB-6008, National Instruments, Austin, TX, USA) sampled voltage signals for further calculation. The order of fluorescence intensity is obtained by linear fitting after logarithm of excitation power and fluorescence intensity, which reflects the proportion of linear and nonlinear components in fluorescence. When exploring the influence of temperature on power dependence, the power dependence at each temperature was measured three times independently, and the mean and standard deviation were calculated.

### 2.7. Anti-Stokes Fluorescence Lifetime Measurement

The anti-Stokes fluorescence lifetime was measured via a time-correlated single-photon counting (TCSPC) system. A 980 nm femtosecond pulsed laser beam was introduced into an inverted microscope. The inside optical path was similar to Section 2.5. Finally, the anti-Stokes fluorescence signals were extracted and detected by an avalanche photodiode (τ-SPAD, PICOQUANT, Germany). The computer with an integrated TCSPC module (DPC-230 Photon Correlator, Becker & Hickl GmbH, Berlin, Germany) was used to record the fluorescence lifetime of samples based on the synchronous signals output by the femtosecond laser and electrical signals from the τ-SPAD. The fluorescence lifetime is equal to the time when the fluorescence intensity decreases from the peak to one of 1/e (e is the base of the natural logarithm).

### 2.8. Optical Setup for Photobleaching Test

A 980 nm excitation light from a continuous-wave semiconductor laser was collimated and then expanded by a lens. In addition, ground glass was introduced to uniformly illuminate the flat cuvette containing BNP-BBTD dots. The anti-Stokes fluorescence was collected by a prime lens (focal length = 35 mm, Tekwin, China) and passed through a 700 nm long-pass filter and a 900 nm short-pass filter. After that, a wide spectral responsive Si-based camera (GA1280, Tekwin, China) was set to detect the anti-Stokes fluorescence.

## 3. Results and Discussion

### 3.1. Characterizations of BPN-BBTD

**Figure 1:**
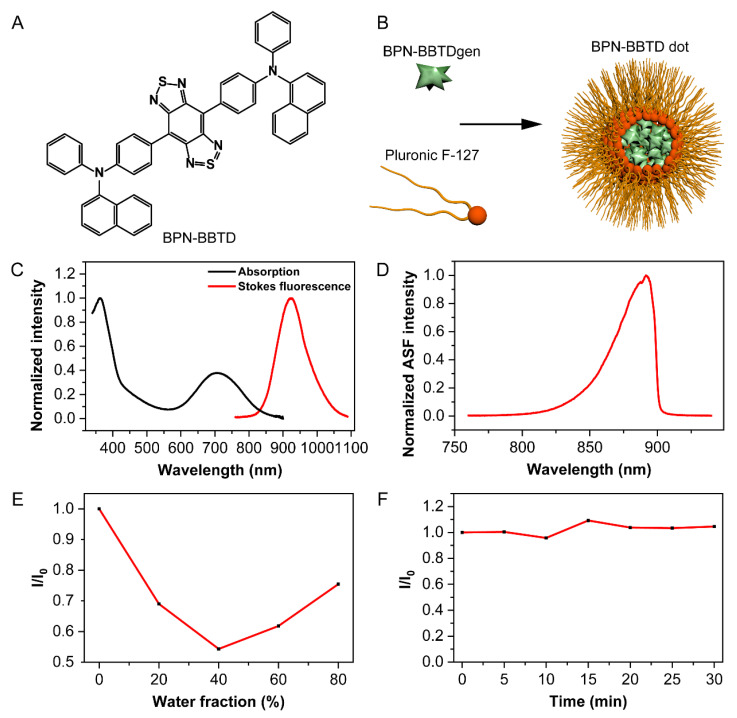
Characterizations of BPN-BBTD. (**A**) The chemical structure of the BPN-BBTD molecule. (**B**) The schematic illustration of the modification of BPN-BBTD dot. (**C**) The normalized absorption and the Stokes fluorescence spectra. The excitation wavelength for Stokes fluorescence is 665 nm. (**D**) The normalized anti-Stokes fluorescence spectrum of BPN-BBTD dots in an aqueous dispersion excited by a 980 nm continuous-wave laser. (**E**) The anti-Stokes fluorescence intensity of BPN-BBTD versus the water fraction in the THF/water mixture. I_0_ and I are the anti-Stokes fluorescence intensities of BPN-BBTD molecules in pure THF and THF/water mixtures with specific water fractions, respectively. The concentration of BPN-BBTD is 2 × 10^−5^ M. The excitation wavelength is 980 nm. (**F**) The anti-Stokes fluorescence intensity of BPN-BBTD dots (2 mg/mL) under the continuous laser irradiation (980 nm, ~500 mW/cm^2^). I_0_ and I are the anti-Stokes fluorescence intensities of BPN-BBTD dots under zero irradiation and after a certain time of irradiation, respectively.

As reported in our previous work [32], BPN-BBTD is an AIE dye with bright near-infrared fluorescence. The molecular structure is presented in Figure 1A. The twisting phenyl/naphthyl rings restrict the intramolecular motion and increase the fluorescence intensity when molecules are in the aggregate state. In order to increase the biocompatibility of the BPN-BBTD dyes, a type of amphiphilic polymer, F127, approved by the US Food and Drug Administration, was used to encapsulate dyes into hydrophilic dots (Figure 1B). As shown in Figure 1C, there is an overlap between the absorption and Stokes fluorescence spectra of the BPN-BBTD dots. Moreover, the BPN-BBTD had two absorption peaks at 360 nm and 706 nm, and an emission peak at 924 nm. We obtained near-infrared fluorescence images of BPN-BBTD dots in aqueous dispersion (Figure S1) using a commercial fluorophotometer as 350 nm, 400 nm, 450 nm, and 500 nm light sources, which further verified that it did have absorption at short wavelength. The small Stokes shift of BPN-BBTD dots held the possibility of thermally activated delayed fluorescence or HBA-induced anti-Stokes fluorescence. Then, a 980 nm continuous-wave laser was chosen instead of the 665 nm continuous-wave laser to excite the BPN-BBTD dots. As shown in Figure 1D, the anti-Stokes fluorescence spectrum between 700 and 900 nm was recorded.

Then, the AIE property of anti-Stokes fluorescence was investigated by changing the proportion of water in the THF/water mixture. As shown in Figure 1E, the anti-Stokes fluorescence intensity initially decreased with an increase in the proportion of water in the THF/water mixture. When the fraction of water exceeded 40%, the anti-Stokes fluorescence intensity increased with the increase of water fraction. This indicated that the anti-Stokes fluorescence of BPN-BBTD dots had AIE properties under the excitation of the continuous-wave laser. The photostability of BPN-BBTD dots in water was further evaluated by a continuous 980 nm laser irradiation for 30 min with a relatively large power density of ~500 mW/cm^2^. It was worth noting that the anti-Stokes fluorescence intensity negligibly reduced during irradiation (Figure 1F).

### 3.2. Mechanism of Anti-Stokes Fluorescence in BPN-BBTD Dots and Its Effect on Nonlinear Optics

**Figure 2:**
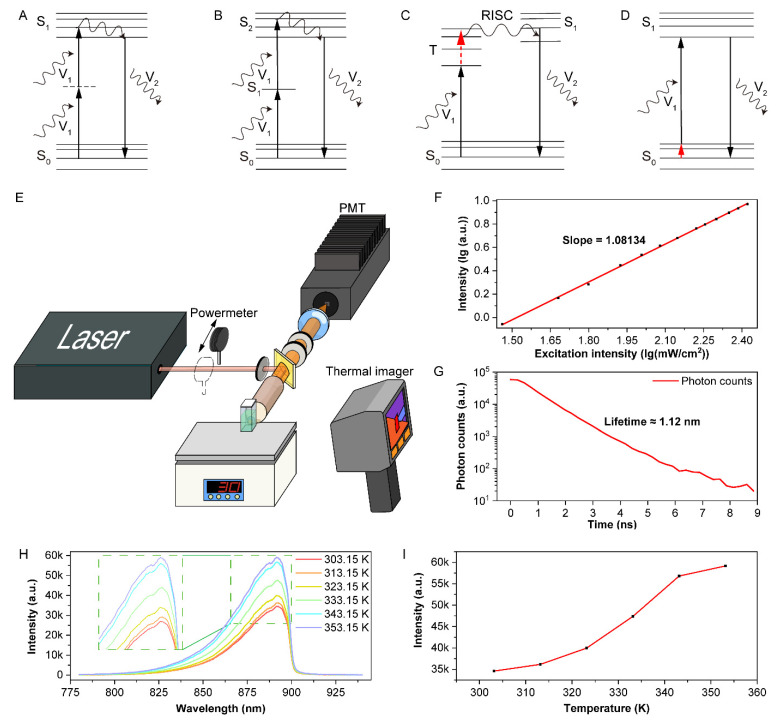
Mechanism of anti-Stokes fluorescence in BPN-BBTD dots. (**A**–**D**) Schematic illustrations of typical anti-Stokes fluorescence processes. (**A**) Two-photon absorption process. (**B**) Upconversion process based on multi-step absorption through intermediate energy levels. (**C**) Thermally activated delayed fluorescence process. (**D**) HBA process. (**E**) Optical setup for the power dependence measurement at various temperatures. (**F**) The logarithmic plot of anti-Stokes fluorescence intensity versus excitation light power (980 nm continuous-wave laser). The black squares and the solid line show the raw data and the fitted curve, respectively, showing a linear dependence of slope 1.08134. (**G**) The photon counts were plotted as a function of time under the 980 nm fs excitation, the lifetime of anti-Stokes fluorescence was about 1.12 ns. (**H**) anti-Stokes fluorescence spectra of BPN-BBTD dots in an aqueous dispersion at different temperatures. (**I**) The variation of anti-Stokes fluorescence peak intensity at different temperatures under a 980 nm excitation.

There are four typical energy-conversion mechanisms to generate anti-Stokes fluorescence, according to the literature [32]. The multi-photon absorption (MPA) process is shown in Figure 2A, in which process molecules simultaneously absorbed two or more low-energy photons to emit one high-energy photon. A typical mechanism of the lanthanide-doped upconversion process is shown in Figure 2B, in which lanthanide ions absorb two or more low-energy photons in sequence to emit one high-energy photon. On one hand, both multi-photon absorption and upconversion processes present a power dependence value greater than one [21,31,33,34]. On the other hand, in thermally activated delayed fluorescence (Figure 2C) or HBA (Figure 2D) processes, one low-energy photon and additional heat energy are absorbed to emit one high-energy photon, indicating the linear power dependence of anti-Stokes fluorescence intensity. To explore the mechanism of anti-Stokes fluorescence of BPN-BBTD dots, we measured the power and temperature dependences, as well as the lifetime of anti-Stokes fluorescence. A system for measuring power dependence and spectra with a temperature controller was built for later experiments, as shown in Figure 2E. Under the excitation of a 980 nm continuous-wave laser, the anti-Stokes fluorescence intensity of BPN-BBTD dots versus the excitation power density was recorded in a logarithmic plot (Figure 2F) in a linearly dependent manner. As shown in Figure 2G, the lifetime of anti-Stokes fluorescence was calculated to be about 1.12 ns. The anti-Stokes fluorescence spectra of BPN-BBTD dots were measured at different temperatures (Figure 2H), and the peak intensities of spectra at different temperatures are recorded in Figure 2I. It could be observed that the anti-Stokes fluorescence intensity significantly increased along with the increase of temperature from 303.15 K to 353.15 K, and the same trend occurred in the second measurement (Figure S2). The relationship between anti-Stokes fluorescence and temperature can be explained by the Boltzmann distribution:

(1) $$\frac{n_{i}}{n_{0}}=e^{-E_{i}/k_{B}T}$$

where $n_{0}$ and $n_{i}$ are the molecular population of the lowest vibrational energy level and higher vibrational energy level $E_{i}$ in the ground state, respectively. $k_{B}$ is the Boltzmann constant, and T is the absolute temperature. As the temperature increases, more molecules are in $E_{i}$, and fewer are in the lowest energy level of the ground state, which enhances the HBA-induced anti-Stokes fluorescence [14,35,36]. According to the above, we concluded that the anti-Stokes fluorescence of BPN-BBTD dots under the excitation of the 980 nm continuous-wave laser was induced by the HBA process.

**Figure 3:**
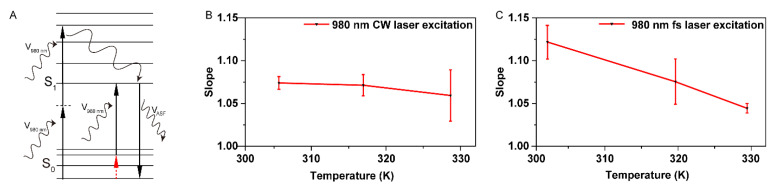
Effect of HBA-induced anti-Stokes fluorescence on nonlinear optics. (**A**) Schematic illustrations of hot-band and two-photon absorption processes of BPN-BBTD dots. The anti-Stokes fluorescence could be produced by both processes. (**B**) The temperature and excitation light power dependence of anti-Stokes fluorescence intensity under the excitation of continuous-wave lasers. (**C**) The temperature and excitation power dependence of anti-Stokes fluorescence intensity under the excitation of femtosecond lasers. Results are presented as mean ± SEM, *n* = 3.

Considering the non-negligible absorption of BPN-BBTD dots between 350 nm and 600 nm (Figure 1C), it is possible that BPN-BBTD dots could absorb two 980 nm photons at the same time (Figure 3A). Although the power dependence values were always about 1, even as the temperature rose from 306 K to 341 K (Figure 3B), reconfirming that BPN-BBTD dots absorbed only one photon under the excitation of a 980 nm continuous-wave laser. The femtosecond laser could compress photons in time to greatly improve the excitation photon density, which promoted the two-photon absorption (2PA) process. To further investigate the relationship between HBA and two-photon absorption, the 980 nm femtosecond laser was chosen to excite BPN-BBTD dots. The power dependence values exceeded 1.10 at 302 K (Figure 3C), which indicated that there might be some two-photon absorption processes mixed with the HBA process. As the temperature increased from 302 K to 329 K (as seen in Figure 3C), the power dependence values obviously decreased, suggesting that the ratio of fluorescence induced by two-photon absorption declined in total anti-Stokes fluorescence, which was reasonable since the increasing temperature promoted the HBA process. These results inspired us to further consider whether the HBA process was involved in typical MPA processes, which could be easily overlooked.

### 3.3. In Vivo Anti-Stokes Fluorescence Confocal Microscopic Imaging

**Figure 4:**
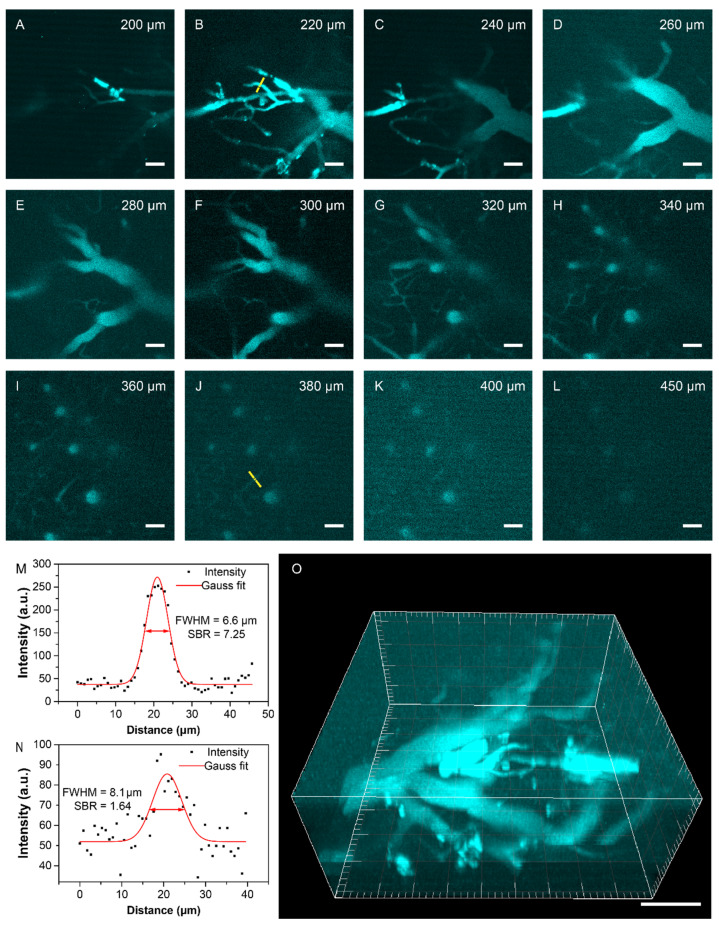
In vivo anti-Stokes fluorescence confocal microscopic imaging. (**A**–**L**) In vivo anti-Stokes fluorescence confocal microscopic images of brain blood vessels at various depths following the injection of BPN-BBTD dots (2 mg/mL, 200 μL) under 980 nm continuous-wave laser excitation. Scale bar: 50 μm. (**M**,**N**) are the intensity distributions along the yellow lines in (**B**,**J**), respectively. The Gauss fits are shown in red curves. (**O**) The 3D reconstruction of a mouse’s cerebral vascular network with 450 μm depth. Scale bar: 100 μm.

To investigate the application of anti-Stokes fluorescence in bioimaging, we used BPN-BBTD dots in bioimaging via a lab-built confocal microscopic system in mice. The imaging system was specially customized to utilize 980 nm excitation and collect 700–900 nm emission fluorescence. Several mice with exposed brains were imaged on the system. Figure 4A–L demonstrates some of the imaging results at different depths. The anti-Stokes fluorescence confocal imaging had an excellent sectioning ability, and cerebral vessels could still be distinguished even at a depth of 450 μm. In addition, we analyzed the signal-to-background ratios (SBRs), and the full widths at half-maximum (FWHMs) of the selected vessels, which are marked in yellow lines in the images of 220 μm and 380 μm depth. As shown in Figure 4M,N, the SBRs were measured to be as high as 7.25 and 1.64, while the FWHMs were calculated to be 6.6 μm and 8.1 μm at those two depths, respectively. Meanwhile, we managed to reconstruct the 3D structure of the cerebral vascular network (Figure 4O).

## 4. Conclusions

Due to the long-wavelength excitation and low autofluorescence, anti-Stokes fluorescence can be effectively utilized in deep-penetration bioimaging. Among five mechanisms of anti-Stokes fluorescence, the HBA-induced process is ignored though it has the advantages of requiring no heavy-metal ions, a short luminescence lifetime, and a high excitation efficiency under a moderate continuous-wave laser. However, the aggregation-induced quenching of common dyes limits the application of HBA-induced anti-Stokes fluorescence in bioimaging. In contrast, AIE dyes have the advantages of bright fluorescence and high photostability when encapsulated into dots, which is suitable for in vivo applications. In this work, the AIE dye BPN-BBTD was proved to have bright anti-Stokes fluorescence. The linear power dependence and the short fluorescence lifetime indicate that the anti-Stokes fluorescence of BPN-BBTD dots is based on the HBA-process under 980 nm continuous-wave laser excitation. Additionally, the two-photon absorption process under the 980 nm femtosecond laser excitation could be weakened due to the increased HBA effect as temperatures increased. Additionally, the anti-Stokes fluorescence confocal microscopic imaging of mice’s cerebral vasculatures was conducted under a 980 nm excitation. With the largest depth of 450 μm, the vasculature network was successfully reconstructed. To our best knowledge, this is the first report of HBA-induced anti-Stokes fluorescence with AIE properties and its application in intravital confocal microscopic imaging with a large depth. We believe that our work provides novel insight into HBA-induced anti-Stokes fluorescence confocal imaging for future research.

## Data Availability

Not applicable.

## Supplementary Materials

The following are available online at https://www.mdpi.com/article/10.3390/bios11110468/s1: Figure S1: Stokes fluorescence macroscopic images of BPN-BBTD dots (25 μg/mL) aqueous solution at different excitation wavelengths; Figure S2: The second measured anti-Stokes fluorescence spectra of BPN-BBTD dots aqueous solution at different temperatures.
