# Supplementary material for: Hot-Band-Absorption-Induced Anti-Stokes Fluorescence of Aggregation-Induced Emission Dots and the Influence on the Nonlinear Optical Effect
Source: Biosensors (Basel). 2021 Nov 22;11(11):468. doi: 10.3390/bios11110468 (PMC8615853; doi:10.3390/bios11110468)
Supplement: Supplementary file 1 [file biosensors-11-00468-s001.zip › biosensors-1423672-supplementary.pdf]

Supplementary Materials

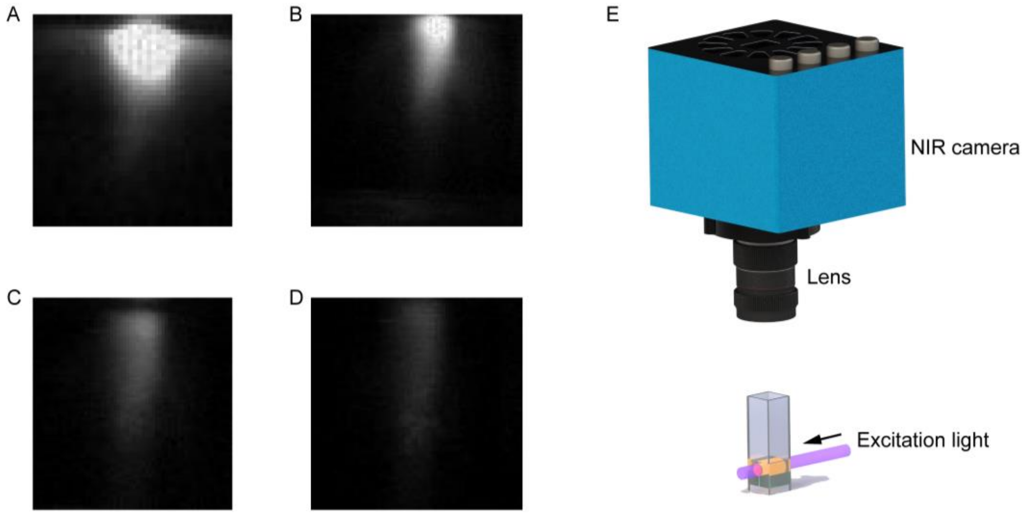

Figure S1. Stokes fluorescence macroscopic images of BPN-BBTD dots (25 μg/mL) aqueous solution at different excitation wavelengths. (A), (B), (C), and (D) are fluorescence imaging (emission > 700 nm) excited by 350, 400, 450, and 500 nm light, respectively. (E) The optical setup of the imaging system. The short-wavelength excitation light beam was directed horizontally into the cuvette and a columnar fluorescence from BPN-BBTD dots (25 μg/mL) aqueous solution was excited. The fluorescence was collected by the lens (focal length = 35 mm, Tekwin, China) above and detected by the NIR camera (SW640, Tekwin, China) after using a 700 nm long-pass filter (FELH0700, Thorlabs) for filtering out the short-wavelength light. Due to the large absorption of BPN-BBTD near 360 nm, the excitation light entered the cuvette and was quickly absorbed, thus forming a bright spot as shown in Figure S1A. When the wavelength was changed to 500 nm, the excitation light penetrated the aqueous dispersion and formed a beam due to the lower absorption.

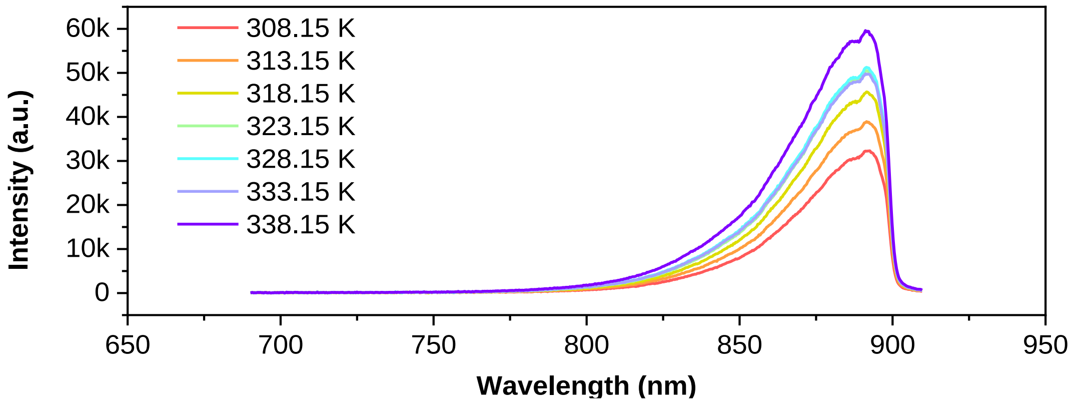

Figure S2. The second measured anti-Stokes shift fluorescence spectra of BPN-BBTD dots aqueous solution at different temperatures.
